# Biophysical aspects underlying the swarm to biofilm transition

**DOI:** 10.1126/sciadv.abn8152

**Published:** 2022-06-15

**Authors:** Vasco M. Worlitzer, Ajesh Jose, Ilana Grinberg, Markus Bär, Sebastian Heidenreich, Avigdor Eldar, Gil Ariel, Avraham Be’er

**Affiliations:** 1 Department of Mathematical Modelling and Data Analysis, Physikalisch-Technische Bundesanstalt Braunschweig und Berlin, Abbestrasse 2-12, D-10587 Berlin, Germany.; 2 Department of Mathematics, Bar-Ilan University, 52900 Ramat Gan, Israel.; 3 Zuckerberg Institute for Water Research, The Jacob Blaustein Institutes for Desert Research, Ben-Gurion University of the Negev, Sede Boqer Campus, 84990 Midreshet Ben-Gurion, Israel.; 4 The Shmunis School of Biomedicine and Cancer Research, Faculty of Life Sciences, Tel Aviv University, Tel Aviv, Israel.; 5 Department of Physics, Ben-Gurion University of the Negev, 84105 Beer Sheva, Israel.

## Abstract

Bacteria organize in a variety of collective states, from swarming—rapid surface exploration, to biofilms—highly dense immobile communities attributed to stress resistance. It has been suggested that biofilm and swarming are oppositely controlled, making this transition particularly interesting for understanding the ability of bacterial colonies to adapt to challenging environments. Here, the swarm to biofilm transition is studied in *Bacillus subtilis* by analyzing the bacterial dynamics both on the individual and collective scales. We show that both biological and physical processes facilitate the transition. A few individual cells that initiate the biofilm program cause nucleation of large, approximately scale-free, stationary aggregates of trapped swarm cells. Around aggregates, cells continue swarming almost unobstructed, while inside, trapped cells are added to the biofilm. While our experimental findings rule out previously suggested purely physical effects as a trigger for biofilm formation, they show how physical processes, such as clustering and jamming, accelerate biofilm formation.

## Teaser

Bacterial swarming ends because of a few cells that initiate the biofilm program, leading to scale-free stationary aggregates.

## INTRODUCTION

Bacteria organize in a variety of collective states, which are referred to as bacterial lifestyles. Transitions between lifestyles, in which the entire bacterial populations change their properties—both at the individual and colony-wide collective levels—are quintessential examples of adaptation to environmental signals and stresses (*1*–*6*). Biofilms are one of the most studied lifestyles, in which multicellular aggregates of bacteria are bound, both to each other or to surfaces, by an extracellular polymeric substances (EPSs) that they secrete (*7*, *8*). Formation of biofilm can occur under different conditions. Correspondingly, transitions to a biofilm lifestyle may take place starting from a variety of other lifestyles such as planktonic (free-floating) bacteria or dense colonies and from a variety of environments, e.g., liquid bulk, surface-attached cells, and wet habitats or dry ones. Here, we consider the less-studied transition from an active swarm to a biofilm. Bacterial swarming is a collective mode of motion in which cells move rapidly over surfaces, forming dynamic patterns of whirls and jets (*9*–*11*). It has been suggested that biofilm and motility, specifically swarming, are oppositely controlled (*12*), making this transition particularly interesting.

Transitions into a biofilm involve a range of cellular changes such as changes in gene expression, motility regulation, and secretion of EPS (*7*, *12*). The stages of these biological processes, in which bacteria switch states, can be referred to as a biofilm program (*4*, *7*, *8*). At the same time, the changing mechanical properties of cells and their environment impose new physical constraints on cells, leading to new dynamical patterns and possibly phase transitions (in the statistical physics sense) (*13*, *14*). The intricate interactions between the biological process involved in the biofilm program and the physics of the bacterial collectives are largely unknown. In particular, it is not clear which effect precedes the other (*12*). It is known that the production of EPS results in cell cohesion and eventual immobility of all cells (*15*). From this perspective, a biological process leads to a change in mechanical cell properties and new physical conditions. On the other hand, it has been suggested that high cell densities lead to jamming and, possibly, phase separation (*16*). Physical theories, for example, the well-studied motility-induced phase separation (MIPS), predict that cells may self-segregate into two phases: a low-density phase of freely moving bacteria (an “active gas” of self-propelled particles) and high-density aggregates of stuck and immobile (jammed) bacteria (*17*–*19*). In turn, jammed cells that are temporarily stuck experience torque that resists flagellar rotation, which may initiate the biofilm program (*20*). This point of view suggests a reversed story, in which a physical phenomenon, namely, jamming and phase separation, triggers the biological one—biofilm program.

*Bacillus subtilis* is a major model organism for studying both biofilm formation and swarming (*8*, *10*, *21*). An emerging view of this organism holds that biofilm formation and motility (be it swimming or swarming) are carefully regulated to be mutually exclusive at the single-cell level (*12*). Biofilm formation in *B. subtilis* results from the activation of several operons devoted to the formation of matrix components including exopolysaccharide (made by enzymes encoded by the *eps* operon) and the protein matrix components TapA and TasA (coded by a second gene operon) (*22*). These two operons and others are regulated by a complex regulatory network, involving quorum sensing (*23*), the DegU regulator, which directly affects swarming motility (*24*), the Spo0A stress master regulator (*25*), and the SinI-SinR-SlrR regulatory pathway (*26*–*28*). In this pathway, SinI acts as an inhibitor of SinR that represses biofilm formation. SinR and SlrR form a complex genetic bistable switch between biofilm formation and motile lifestyle (either swarming or swimming). The mutual exclusive nature of motility and biofilm formation is also enhanced by the *epsE* gene (part of the EPS operon), whose product acts as a clutch that prevents flagellar rotation upon activation of the biofilm program (*29*). The details of the biofilm program were studied in nonswarming (e.g., planktonic) populations. However, the role of EPS at the terminal stages of swarming is not clear.

MIPS (*17*–*19*) has been suggested to explain self-segregation of active particles in the absence of signaling or attracting forces. MIPS has been observed experimentally in suspensions of colloidal particles, for example, Janus particles (*30*) or self-propelled rollers (*31*). The main assumption underlying MIPS is that at sufficiently high densities, frequent collisions or jamming of active particles lead to a reduction in the average speed (*18*). This is consistent with measurements of the average cell speed in swarm assays in which the average cell speed increases for low to intermediate densities but decreases at high ones (*32*, *33*). For example, it has been speculated that MIPS underlies the fruiting body formation in *Myxococcus xanthus* (*34*) and the swarming to biofilm transition in *B. subtilis* (*16*). Recently, the standard MIPS theory has been generalized to account for elongated particle shapes (*35*–*37*), hydrodynamic interactions (*38*, *39*), and population growth (*40*, *41*). Such additional effects can lead to arrested phase separation instead of the macroscopic phase separation expected from MIPS, i.e., finite size clusters of jammed particles, baring some resemblance to high-density clusters within swarming colonies. These theoretical studies provide further theoretical support to the MIPS triggering biofilm hypothesis (*41*).

Here, we present a detailed experimental study of the final stages of swarming, right before biofilm formation, in colonies of *B. subtilis*. We analyze the dynamics in the intermediate regions between the colonial center and its edge, where swarming cells coexist with dense stationary aggregates (*13*). Accordingly, we start with a quantitative analysis of the movement statistics at different stages of the swarm to biofilm transition. We show that some of the main assumptions underlying MIPS do not hold. In particular, the speed of active cells is always increasing with the local density. Next, we demonstrate that trajectories of individual cells are composed of super- and subdiffusive regimes as cells continuously enter or leave aggregates, indicating a (partially reversible) trapping scenario. In addition, experiments with biofilm-deficient mutants that do not produce any biofilm components show that stable stationary aggregates are not possible without these components. Last, we use a mutant that fluoresces upon transitioning into the biofilm state to illustrate how a subpopulation of cells initiates the formation of stationary aggregates. Therefore, we conclude that a biophysical mechanism leads to trapping of motile cells, to the formation of stationary aggregates, and eventually to stable biofilms.

## RESULTS

Following inoculation and an initial lag phase, the colony starts expanding, eventually covering the entire agar plate. The front of the colony is a highly active monolayer of swarming cells, moving rapidly in so-called rafts or dynamic clusters (*13*). Cells at the center are nonmotile and are arranged in a multilayered structure, constituting a biofilm. As the colony expands, the biofilm constituting the center of the colony grows radially as well, typically much slower than the expanding front. See (*13*) for a detailed and comprehensive study of the spatiotemporal dynamics of an expanding colony of swarming *B. subtilis*.

### Dynamics in the coexistence zone

**Fig. 1.**
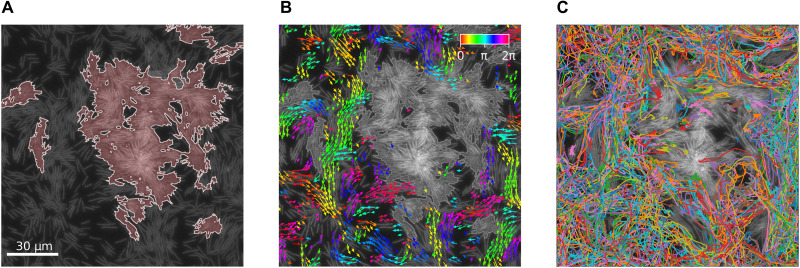
Coexisting swarming and stationary cell aggregates. (**A**) Stationary aggregates of cells are highlighted in red. (**B**) Bacterial flow field obtained from optical flow. No flow is plotted inside stationary aggregates. Color indicates orientation. (**C**) Individual trajectories obtained by tracking cells superimposed on microscopic image.

We start by studying the swarming dynamics during the expansion phase, at intermediate regions between the colony center and its edge, where coexistence of swarming cells and stationary aggregates is found. Figure 1A shows stationary aggregates of cells, which are large connected regions with very low speeds. These aggregates are surrounded by swarming cells moving at significantly higher speeds. Figure 1B shows the coarse-grained velocity of the swarming regions, obtained by optical flow analysis [see (*42*) and section S2.1]. The distinction between the regions is a threshold speed. In addition, we analyze the trajectories of individual cells (see Materials and Methods), as depicted in Fig. 1C. The trajectories reveal that cells enter and leave the stationary aggregates, intermittently. Therefore, the composition of the aggregate is not fixed, and aggregate boundaries are dynamic—slowly moving, merging, and splitting.

**Fig. 2.**
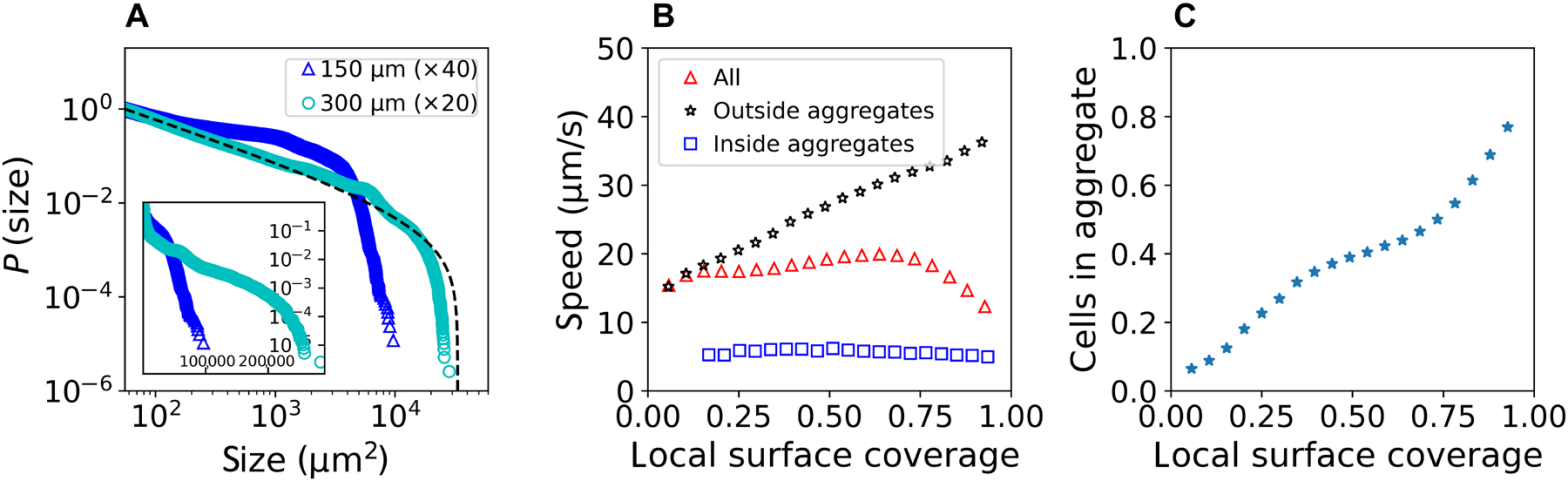
Movement statistics. (**A**) ASD. The complementary cumulative distribution *P*(*x*) for fields of view with side lengths of 150 μm (blue triangles, ×40 magnification) and 300 μm (cyan circles, ×20 magnification). As a visual guide, a fit to a power law with exponential cutoff at ×20 is shown (dashed line). Inset shows the same data on a semilog scale. Data are obtained by averaging over several independent experiments (details in Materials and Methods and the Supplementary Materials). (**B**) Average speed as a function of local surface coverage. The speed is obtained by binning and averaging over bins with similar surface coverage. Shown is the average speed of all cells (red triangles), outside stationary aggregates (black stars), and inside stationary aggregates (blue squares). Data are obtained by averaging over several independent experiments. The global surface coverage varies between 0.5 and 0.8 for all experiments. The averaged local surface coverage inside the aggregates is on average a factor of two larger than outside. (**C**) Average percentage of cells within a bin that belong to a stationary aggregate. The plot is obtained from the same data as in (B).

Quantitatively, Fig. 2A shows the distribution (via the empirical complementary cumulative distribution function) *P*(*x*) of the aggregate sizes, i.e., the aggregate size distribution (ASD), at two different magnifications. The ASD appears to obey a power law over several orders of magnitude. Using a maximum likelihood fitting and the Akaike information criterion (AIC) (*43*), we find a power law with exponential cutoff, $P(x)=C\int_{x}^{\infty }r^{-\alpha }e^{-\lambda r}\;\mathrm{dr}$ with α ≈ 1.9 and λ ≈ 2 × 10^−5^, where *C* is a normalization constant. A power law with exponential cutoff with similar exponents was also found to describe area size distribution of moving clusters in the “swarming” phase of several bacterial species specifically *B. subtilis* (*44*) and *M. xanthus* (*45*, *46*) at lower densities. A simple kinetic mean-field approach reproduces such power laws with exponential cutoff. The exponents of ASD were found to be nonuniversal and depend on the loss rates of bacteria from clusters and the growth rates of clusters by fusion (*47*). The AIC alternatives in our analysis here were taken to be pure power laws and pure exponential distributions (section S2.3). The cutoff is related to finite size effects and depends on the size of the viewing field: At ×40 magnification, the cutoff point is much lower compared to the cutoff point at ×20 magnification. This indicates that larger stationary aggregates cannot be resolved by the finite observation window. Testing this hypothesis by further decreasing the magnification is technically difficult as the dynamical data needed to define stationary aggregates become less reliable at smaller magnifications. Overall, these findings indicate that there is no characteristic aggregate size on experimentally accessible scales.

Outside aggregates, cells move freely, showing coexistence of moving and nonmoving cells in the same region. While previous experiments reported a nonmonotonous dependence of the average speed on the density (*32*, *33*), other works have argued that a monotonic reduction of speed with increased density is as an explanation for clustering of active particles (*18*, *19*). Here, we measure the dependence of the average speed on the local surface coverage and compare the average speed in the entire viewing field with the average obtained only inside or outside the stationary aggregates. Considering the entire viewing area, Fig. 2B shows that the average speed is roughly constant for low to intermediate surface coverages, followed by a decrease at high surface coverages. This result is different from previous measurements, e.g., (*14*, *32*, *33*), in which the average speed is significantly increasing at low to medium coverages but decreasing at high ones. This is due to two reasons: (i) Here, samples are taken closer to the colony center, a region that has already started transforming to a biofilm. (ii) The definition of surface coverage is a bit different. Here, it refers to local coverage (see section S2.2 for details). In addition, the coverage can get very high (practically up to 1) because we are sampling local highly dense aggregates that may be starting to become multilayered. In contrast, Be’er *et al.* (*14*), Sokolov *et al.* (*32*), and Ariel *et al.* (*33*) considered the average coverage in the entire viewing area.

On the basis of the distinction between swarming regions and stationary aggregates, we estimate the dependence of the average speed on surface coverage in each region separately. We find that outside stationary aggregates, the average speed of freely swarming cells is always increasing with surface coverage, close to linearly. Unexpectedly, this holds up to very high densities (practically 1). Again, note that speed and density are calculated locally (section S2.2), so the measurement excludes jammed aggregates. In contrast, the speed inside stationary aggregates remains fairly constant at a low value, independent of the surface coverage. Hence, the apparent nonmonotonous dependence of the average speed on surface coverage for all cells is due to the different fraction of cells in the two regions, see Fig. 2C. In other words, the reduction of speed at high surface coverage results from a higher fraction of cells belonging to stationary aggregates and not from slowing down of moving cells.

**Fig. 3.**
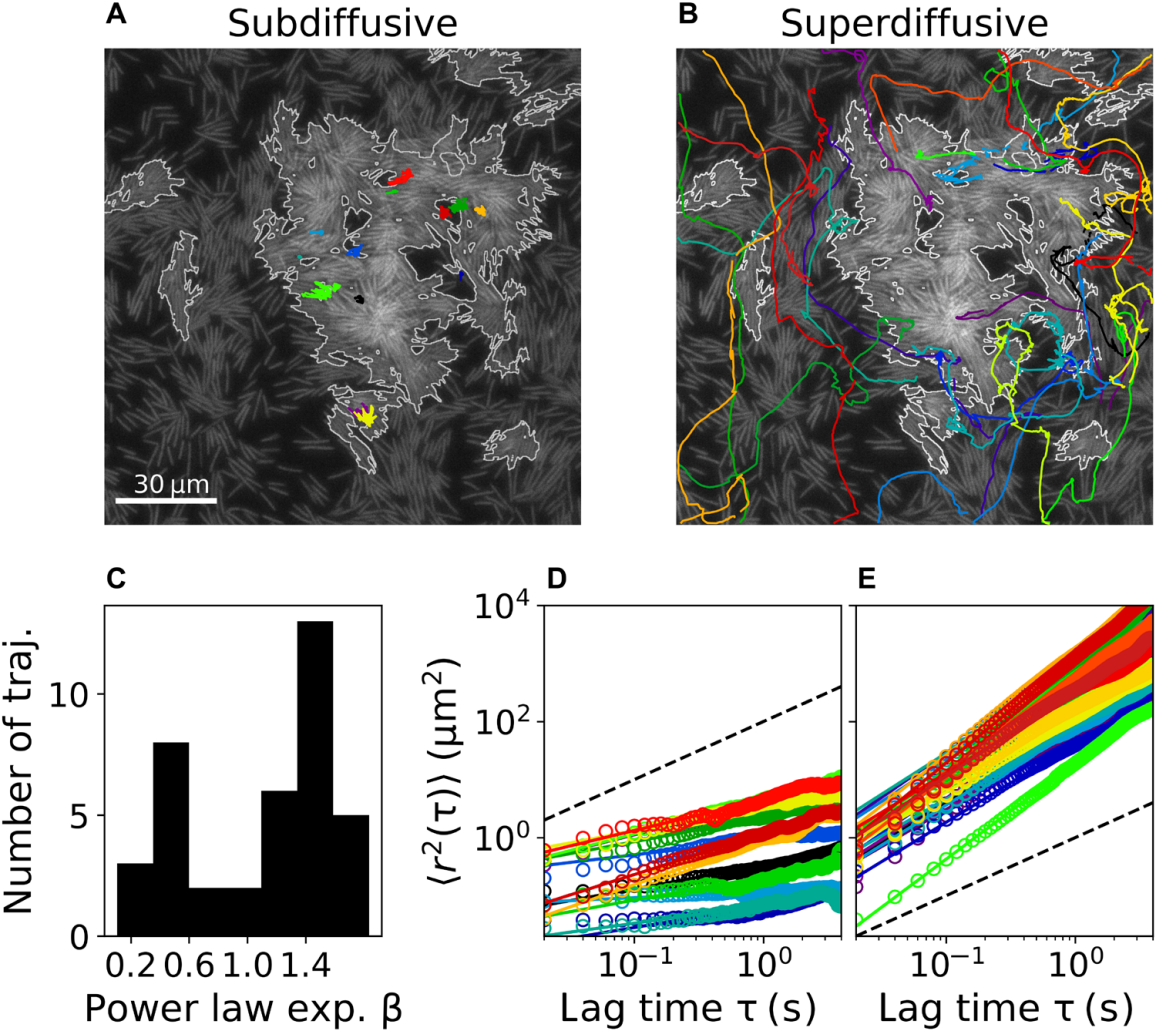
Trajectory analysis. (**A**) Subdiffusive and (**B**) superdiffusive trajectories superimposed on the microscopic image at *t* = 0, while stationary aggregates are outlined. (**C**) Histogram of power law exponents β obtained from nontransitive trajectories. Mean square displacements (open circles) as a function of lag time for (**D**) subdiffusive trajectories [color matches the trajectories of (A)] and (**E**) superdiffusive trajectories [color matches the trajectories of (B)]. Fits are provided as solid lines, while normal diffusion is indicated as a dashed line.

On the individual scale, we study the trajectories of single bacteria within the collective (see Fig. 3, A and B). To do so, individual cells are tracked to obtain their trajectories, denoted by *r*(*t*). The mean square displacement is computed, <*r*^2^(τ)> = <∣*r*(*t*) − *r*(*t* + τ)∣^2^>, and fitted to a power law, <*r*^2^(τ)> ∼ τ^β^. See section S2.4 for details. Figure 3C shows a bimodal distribution of exponents β, related to either subdiffusive (β < 1; see Fig. 3D) or superdiffusive (β > 1; see Fig. 3E) trajectories. Moreover, overlaying the trajectories with the collective motion shows that subdiffusive behavior is found inside stationary aggregates (Fig. 3A), while superdiffusion is most commonly observed outside of stationary aggregates (Fig. 3B). A few trajectories show diffusive or transitive dynamics (i.e., sub- or superdiffusive on different time scales). Manual inspection showed that such trajectories correspond to bacteria that entered or left aggregates during the periods that they were tracked.

Overall, the analysis of trajectories suggests the following microscopic picture: Within the swarming regions, cells are superdiffusive, in accordance with previous results, showing that trajectories of individual swarming cells are consistent with Lévy walks (*48*, *49*). In contrast, cells trapped inside stationary aggregates show subdiffusive behavior. Furthermore, there is a slow exchange of cells between the swarm and the aggregates, as observed in transitive trajectories. That is, swarming cells can get trapped in stationary aggregates, while trapped cells can also leave a stationary aggregate and resume swarming.

Next, we study the mechanism leading to the nucleation of large stationary aggregates (i.e., with a size that is comparable to the observed domain). The linear increase in speed with surface coverage for swarming cells indicates that stationary aggregates do not emerge because of jamming at high densities. However, trajectory analysis suggest that trapping is partially responsible to the creation and dynamics of aggregates. We will now show that these observations are related to the biological cellular processes of cells initiating the biofilm program.

### Biophysical origin of the stationary aggregates

**Fig. 4.**
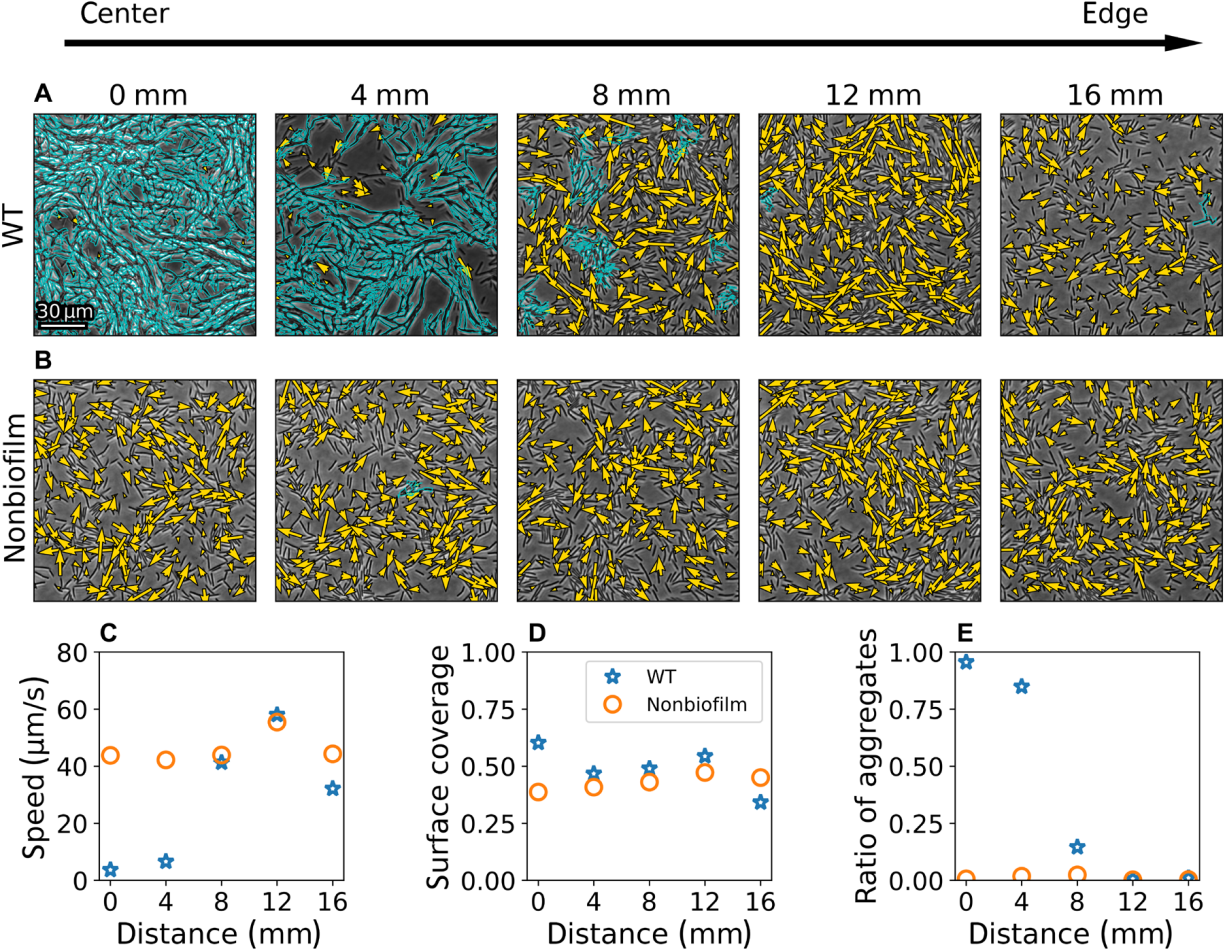
WT versus nonbiofilm mutant. Snapshots of (**A**) the WT and (**B**) the nonbiofilm mutant for a variety of regions from the edge of the inoculum (center) toward the edge of the colony in steps of 4 mm (left to right). Flow is superimposed as in Fig. 1B, while stationary aggregates, detected using optical flow analysis, are encircled in cyan. (**C**) Average speed, (**D**) surface coverage, and (**E**) the ratio of aggregates for the WT (blue stars) and the nonbiofilm mutant (orange circles) at different distances from the edge of the inoculum.

To test the hypothesis that the presence of stationary aggregates is related to biofilm formation, we use a mutant that is defective in the production of biofilms (strain 7871; see Materials and Methods). Hence, we will refer to the mutant as “nonbiofilm mutant.” In particular, the mutant cannot produce EPSs, among other things. Figure 4 (A and B) shows typical snapshots of the dynamics for a wild-type (WT) colony and for the nonbiofilm mutant at different distances from the colony center (edge of the inoculum), respectively. In the WT colony, the center is dominated by densely packed, stationary cells that form a biofilm. Toward the edge of the WT colony, swarming is observed, while between the center and the edge, swarming cells and stationary aggregates coexist. The nonbiofilm mutant shows uniform swarming behavior throughout the entire colony. That is, the dynamics looks similar, independent of the location within the colony. Note that the optical flow algorithm detects a few spurious small stationary aggregates that are probably caused by cell jamming. However, these aggregates do not grow to the large aggregates as observed for the WT.

To quantify the differences between the WT and the nonbiofilm mutant, we measure the average speed, the surface coverage, and the ratio of stationary aggregates at different distances from the center. Following (*13*), studying different spatial regions is equivalent to looking at different times during the colonies expansion, where looking close to the inoculation center is analogous to later times. The ratio of aggregates is defined as the ratio of cells that belong to a stationary aggregate to all cells in the field of view. For the nonbiofilm mutant, there are almost no stationary aggregates, and the speed and density remain fairly constant (see Fig. 4, C to E). For the WT, there are clear differences between the center and the edge of the colony. Close to the center, surface coverage is high, and almost all cells are inside stationary aggregates. Accordingly, the average speed is very low. Toward the colony edge, the statistics are comparable to the nonbiofilm mutant. We remark that the difference in speed for the last data point between the WT and the nonbiofilm mutant is correlated with the difference in surface coverage. Hence, the slightly slower WT speeds can be explained by the slightly lower densities [compare Fig. 2B and (*14*)]. In conclusion, the main difference between the WT and the nonbiofilm mutant is the presence/absence of large stationary aggregates. This supports the hypothesis that the stationary aggregates are related to biofilm formation.

**Fig. 5.**
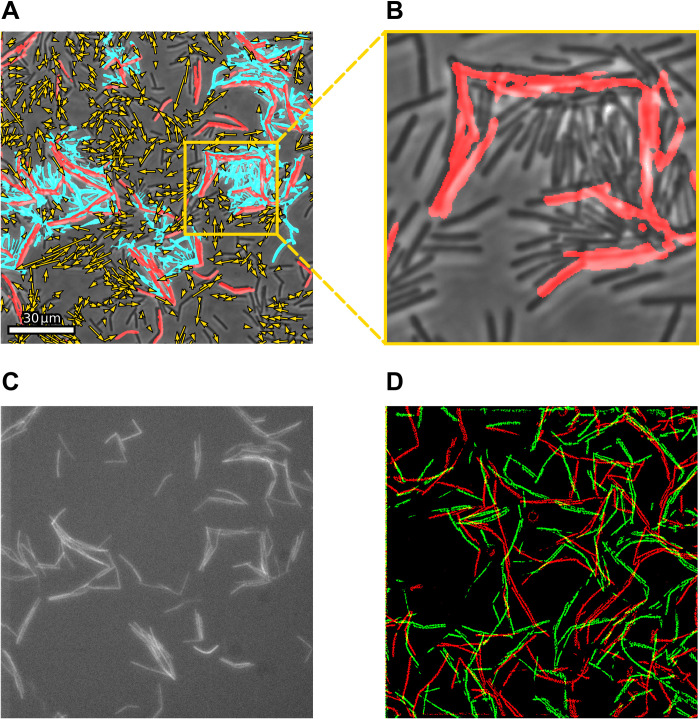
Nucleation of stationary aggregates. (**A**) Snapshots of stationary aggregates. Cells secreting EPS are highlighted in red, and stationary aggregates are shown in cyan. Bacterial flow field is provided by yellow arrows. (**B**) Zoom-in from the highlighted region in (A), showing the trapping of motile cells by EPS-secreting cells. For better visibility, stationary aggregates are not highlighted. (**C**) Raw image of EPS-secreting cells shown in (A). (**D**) EPS-secreting cells for two different times separated by an hour, where red cells represent the earlier time and green cells represent the later time. The field of view shown in (D) is different from the field of view shown in (A) to (C).

It is well known (*2*, *13*) that genetically identical cells within a colony of *B. subtilis* show phenotypic differentiation. In particular, only a fraction of cells produces EPS, increasing cohesion between cells. This suggests EPS as a possible trapping mechanism that nucleates the stationary aggregates. To illustrate this point, we use a strain expressing yellow fluorescent protein (YFP) upon secretion of EPS (strain 1034; see Materials and Methods and Fig. 5, A to C). We observe that cells secreting EPS are immotile. As predicted by simple models of self-propelled particles (*50*), such stationary obstacles can act as particle traps and become seeds for stationary aggregates. However, Fig. 5D shows that EPS-secreting cells might be displaced over time. This may be due to motile cells that push immotile cells around, for example, if sufficiently many cells align to form a moving cluster. Again, such a behavior has been predicted from theory (*51*). Together, we conclude that mechanical and biological mechanisms combine to form stationary aggregates: A subpopulation of cells in the colony is immotile matrix producers. Depending on several factors, for example, the arrangement and alignment of cells or the strength of cell substrate adhesion among others, immotile cells either get rearranged by motile cells or form traps, which nucleate stationary aggregates.

**Fig. 6.**
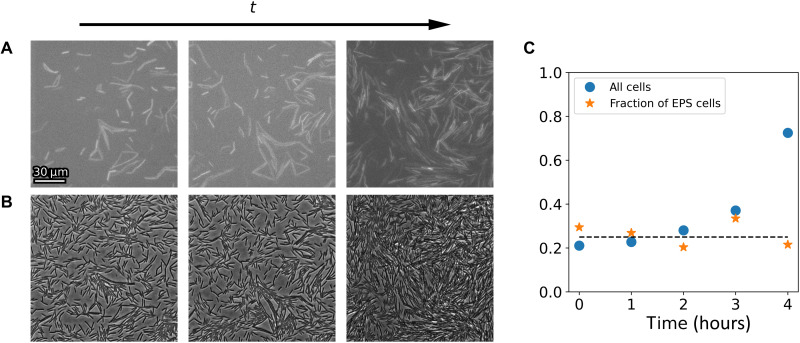
Long-time dynamics. Snapshots of (**A**) EPS-secreting cells and (**B**) all cells over time. Snapshots are taken at the same position at 2-hour intervals. (**C**) Surface coverage of all cells (blue circles) and the fraction of EPS-secreting cells of all cells (orange stars) over time. The dashed line is a visual guide to the eye at 25%.

Figure 6 shows the fraction of EPS-secreting cells out of all cells, as well as all cells in the field of view for different stages along the swarm-to-biofilm transition. The fraction of EPS cells does not increase significantly over time but remains at around 25% of all cells. The overall cell density does increase. This indicates that cells within stationary aggregates not necessarily start producing EPS. This is in line with earlier experimental observation of division of labor within mature biofilms (*3*, *52*–*54*).

## DISCUSSION

Analyzing the dynamics in different zones between the colony edge and its center in a colony of *B. subtilis* allows us to identify some of the fundamental biophysical mechanisms underlying the formation of stationary bacterial aggregates and eventually biofilms. We find that a few cells initiating the biofilm program act as seeds for stationary aggregates. Physical interactions (e.g., mechanical or steric) lead to trapping of swarming cells and to growth of aggregates with power law features over several orders of magnitude. At later stages, most cells within the aggregates transition to biofilm as well. We found that macroscopic phase separation (or arrested aggregation with macroscopic clusters) is not observed without cells transitioning into biofilm. Hence, MIPS does not play a role in the termination of swarming and transition to biofilm. This does not mean that density fluctuations, leading to transient and local creation of small aggregates of temporarily immobile cells are not an important step in triggering the biofilm program in the initial stages of the swarm to biofilm transition.

While the biological principles underlying biofilm formation have been researched extensively, the swarming to biofilm transition is still far from being understood (*12*, *13*, *21*, *55*–*59*). In particular, it is not clear what are the environmental conditions under which a colony “decides” to stop swarming and transition into a biofilm. The present work on *B. subtilis*, which is a well-studied model organism for both swarming and biofilm, addresses this question by considering the transition from the point of view of the end of swarming. Our results indicate that the end of swarming is governed by the interplay between physical and biological processes. This is in contrast to recent purely physical explanations for the end of swarming (*16*).

To our knowledge, the nucleation of stationary aggregates, showing power law–like features, from a bacterial swarm is not predicted by currently available models of bacterial swarms (*5*, *13*, *16*, *38*, *39*, *60*). Several particle-based and coarse-grained continuum models account for certain aspects of this transition, such as a separation into stationary aggregates surrounded by an active swarm phase (*38*, *39*), an increased surface adhesion of cells (*13*) or the transition from a two-dimensional monolayer to three-dimensional structures (*60*). However, none of these models cover the swarm to biofilm transition in its entirety. Modeling bacterial swarms and their internal dynamics is a highly active area of research and beyond the scope of this study. Our results encourage the development of novel models that address the transition from swarming to biofilm.

The biological advantages of the stationary aggregates remain unclear. We suggest two possible scenarios: (i) Stationary aggregates are early forms/precursors of biofilms. During the spread of the colony through swarming, a subpopulation of cells responds to environmental cues, producing EPS. These cells act as founder cells for new biofilms far away from the inoculum. (ii) Stationary aggregates accelerate the spreading of biofilms. A colony of *B. subtilis* intrinsically features subpopulations of swarming cells and EPS-secreting cells. Because of steric interactions, EPS-producing cells are transported away from the inoculum, where they nucleate stationary aggregates. In both scenarios, stationary aggregates assist in colonizing the surface with biofilms. Expansion of biofilms is usually mediated by the growth and division of individual cells within the biofilm. This process is typically slow due to limited local nutrients, leading to suboptimal exploration of the available space. On the other hand, swarming facilitates rapid expansion and surface exploration. Hence, the formation of stationary aggregates may accelerate the spread of biofilms over surfaces on much shorter time scales compared to purely growth mediated expansion. The latter scenario questions the nature of swarming, as it might be a temporary state whose main role is to accelerate the spread of biofilms. Hence, swarming and biofilms are not antagonistic states but evolutionary beneficial to ensure a rapid spreading of sheltered communities in the form of biofilms.

## MATERIALS AND METHODS

All strains were constructed in the background of WT *B. subtilis* strain NCIB3610. To construct new *B. subtilis* strains, standard transformation and SPP1 transduction protocols were used for genomic integration and plasmid transformation. Strains AES4846 and AES4847 (amyE::Pveg_R0_sfGFP;spec^R^ and amyE::Pveg_R0_mKate;spec^R^) expressing constitutive green fluorescent protein (GFP) and red fluorescent protein (RFP), respectively, were constructed to ease cell tracking by corresponding transduction of strains SG13 and SG48 (*61*) into the WT with selection for spectinomycin. We refer to these two strains as WT as they behave similar to the nontagged WT even while the fluorescence-activating light is on, with no photobleaching or changes in motility/statistics, etc. (*62*). Strain AES1034 (Peps-YFP), expressing YFP only upon transition into the biofilm state, was previously described (*23*). Strain AES7871, an RFP-tagged *sinI* mutant [amyE::Pveg(+1/+8) R0mKate2 spec Δ*sinI*:kan] (defective in biofilm components), was constructed by DNA transduction from the *sinI* mutant strain BKK24600 (*63*) into strain AES4847 with selection for kanamycin. Antibiotics were added to each frozen stock and during regular growth in LB liquid medium or on 2% hard agar plates, at the following concentrations: spectinomycin, 100 μg/ml; kanamycin, 10 μg/ml. Swarm experiments were performed without additions of antibiotics. Labeling was stable.

Isolated colonies from hard agar plates were cultured in a 2-ml LB liquid medium for each strain and incubated at 200 rpm and 30°C overnight. The overnight cultures were inoculated to the swarm plates. Swarm plates are standard 8.8-cm petri dishes filled with 15 ml of molten 0.5% agar supplemented with LB. Swarm plates were prepared 20 hours before inoculation and aged at 22°C and 40% relative humidity and then 8 min in the flow hood. Four microliters of culture was inoculated at the center of the plates and allowed to dry for 1 hour before incubation. Swarm plates were then incubated at 30°C until the swarm colony diameter was 4 cm and observed under the optical microscope. To follow trajectories of single cells in a swarm, the two WT strains, green (4846) and red (4847), were mixed, where one constitutes nearly 99% of the population and the other 1%. Mixing was done right before inoculation, in an external vial, after each one was grown separately overnight. This enabled us to track the trajectories of single cells moving among the same population, only that the color distinguishes between them.

An optical microscope (Zeiss Axio Imager Z2) equipped with long-distance phase contrast/fluorescence lenses of 40× and 20× was used to record microscopic swarm dynamics. The movies were captured at 50 frames/s and 900 × 900 pixels for several seconds. These movies streamed directly to the hard disk, resulting in hundreds of images in a sequenced movie. Recorded movies were analyzed in Python using the Farnebäck method (*42*) to obtain the optical flow between consecutive frames. For details on the statistical methods, refer to section S2. To distinguish between two populations (two colors), high-resolution images were taken using an Optosplit II, Andor, hooked to a Zeiss Axio Imager Z2 microscope. The system splits a dually excited image (excitation, 59026x; beam splitter, 69008bs; and emission, 535/30 nm and 632/60 nm) on a NEO camera (900 × 1800 pixels and 50 frames/s) to generate two simultaneous but separate fields of view, green and red, that are then merged again following postprocessing [for more details, see (*62*) and section S2.4].

## Supplementary Materials

### This PDF file includes:

Supplementary Text Figs. S1 to S3 Tables S1 and S2 References
