# Supplementary material for: Biophysical aspects underlying the swarm to biofilm transition
Source: Sci Adv. 2022 Jun 15;8(24):eabn8152. doi: 10.1126/sciadv.abn8152 (PMC9200279; doi:10.1126/sciadv.abn8152)
Supplement: Supplementary file 1 — Supplementary Text Figs. S1 to S3 Tables S1 and S2 References [file sciadv.abn8152_sm.pdf]

Science Advances
AAAS

Supplementary Materials for

Biophysical aspects underlying the swarm to biofilm transition

Vasco M. Worlitzer *et al.*

Corresponding author: Gil Ariel, arielg@math.biu.ac.il; Avraham Be'er,beera@bgu.ac.il

Sci. Adv. **8**, eabn8152 (2022)
DOI: 10.1126/sciadv.abn8152

This PDF file includes:

Supplementary Text
Figs. S1 to S3
Tables S1 and S2
References

S1 Data Acquisition

The data for Fig. 2 is obtained from five individually recorded movies in the transition zone. All movies were captured within ≈ 5 minutes. Three movies were taken at $40\times$ magnification and two movies at 20x magnification. One movie of each magnification is recorded for 5000 frames, whereas the rest is recorded for 2000 frames each. Movies were captured at 50 frames per second.

S2 Data Analysis

S2.1 Flow computation and aggregate identification

The raw image data obtained from the experiments is used to compute the flow and identify different regions as illustrated in Fig. S1. First, the optical flow is computed via the Farnebäck algorithm (*45*), which provides the local velocity field $\mathbf{v}(\mathbf{x}, t)$ shown in Fig. S1**B**. Parameters of the Farnebäck algorithm are provided in table S1. We then identify stationary clusters in four steps: First, the original image is smoothed once with a Gaussian kernel. Then, cells are separated from the background by thresholding the smoothed image. Based on the flow, nearly stationary regions are identified (i.e. regions below a certain magnitude of the flow). Finally, connected stationary regions above a certain size are labeled as a stationary cluster, see Fig. S1**C**. We require stationary aggregates to at least cover the same area as roughly ten bacteria. From our data, we estimate the surface coverage of a typical cell to be roughly 200 pixels (at $40\times$ magnification), hence we set a size threshold of 2000 pixels (at $40\times$ magnification).

S2.2 Binning

To estimate quantities such as the local velocity and speed, we divide each snapshot into 8×8 ($40\times$ magnification) or 16×16 ($20\times$ magnification) bins, see Fig. S2. For each bin, the surface coverage as well as the average speed of cells is measured. Due to the large size of the bins, we averaged the resulting data in Fig. 2**B** over bins with similar surface coverage to reduce fluctuations. To obtain the statistics inside and outside stationary aggregates (see Fig. 2**B**), we either neglect all bins that contain stationary aggregates or only consider bins in which all cells are part of a stationary aggregate respectively.

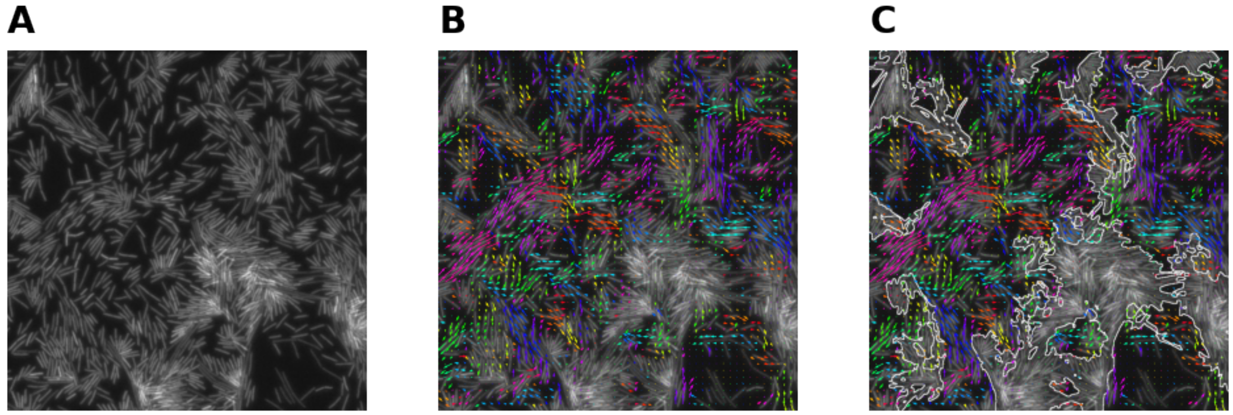

Figure S1: **Identification of stationary aggregates.** **A** Raw image obtained from the microscope. **B** Computed flow superimposed on the raw image. Color code of the arrows represent the angle of the flow, i.e. areas with the same color show cells moving in the same direction. **C** Stationary aggregates, i.e. slow areas of at least 10 bacterial surface coverages, are encircled and plotted along the flow.

Parameter	40×	20×
pyr_scale	0.5	0.5
levels	3	3
winsize	20	10
iterations	3	3
poly_n	5	5
poly_sigma	1.1	1.1
flags	0	0

Table S1: Parameters used for the Farnebäck algorithm (*45*) as implemented in OpenCV (*64*) for 20× and 40× magnification.

Parameter	40×	20×
σ	2	1
min_area	2000	500
min_speed	2	1

Table S2: Parameters used to identify stationary aggregates, i.e. σ (size of the Gaussian kernel used for smoothing), min_area (minimal size of an aggregate in pixels), min_speed (speed threshold for aggregates in pixels per frame).

S2.3 Aggregate size distribution

We assume that the aggregate size distribution (ASD) shown in Fig. 2**A** can be reasonably well approximated by a statistical model. To compare the relative quality of different models, we use the Akaike information criterion (AIC) (*46*). Our choice of models is motivated by the empirical complementary cumulative distribution function of the data. In particular, we consider an exponential distribution

$$f_\lambda(x) = \lambda e^{\lambda(x_{min}-x)}, \tag{S1}$$

a power-law

$$f_\alpha(x) = \frac{\alpha-1}{x_{min}}\left(\frac{x}{x_{min}}\right)^{-\alpha}, \tag{S2}$$

and a power-law with exponential cut-off

$$f_{\alpha,\lambda}(x) = C(x_{min},\lambda,\alpha)x^{-\alpha}e^{-\lambda x} \tag{S3}$$

with

$$C(x_{min},\lambda,\alpha) = \frac{\lambda^{1-\alpha}}{\Gamma(1-\alpha,\lambda x_{min})}, \tag{S4}$$

where $\Gamma(s,x) = \int_x^\infty r^{s-1}e^{-r}dr$ is the upper incomplete gamma function. Note that one can obtain an exponential distribution and a power-law from the the power-law with exponential cut-off in the limits $\alpha \to 0$ and $\lambda \to 0$ respectively. The minimal value x_{min} is given by the threshold value we used to identify aggregates, see SM S2.1. That is, we assume that the statistical model holds for the entire range of the data. Estimates for the parameters λ and α are obtained from a maximum likelihood approach.

Computing the AIC for the data recorded at 20× magnification for the three candidate models reveals that the power-law with exponential cut-off is several times more likely to produce the data than the alternatives. In Fig. 2**A** the tail distribution of a power-law with exponential cut-off, i.e. $P(x) = \int_x^\infty f_{\alpha,\lambda}(r)\,dr$, is shown as a visual guide.

S2.4 Single-cell tracking

To track single cells as well as the collective behavior simultaneously, the colony was prepared as described in section 2. This procedure results in two images, obtained at the same position within the colony at the

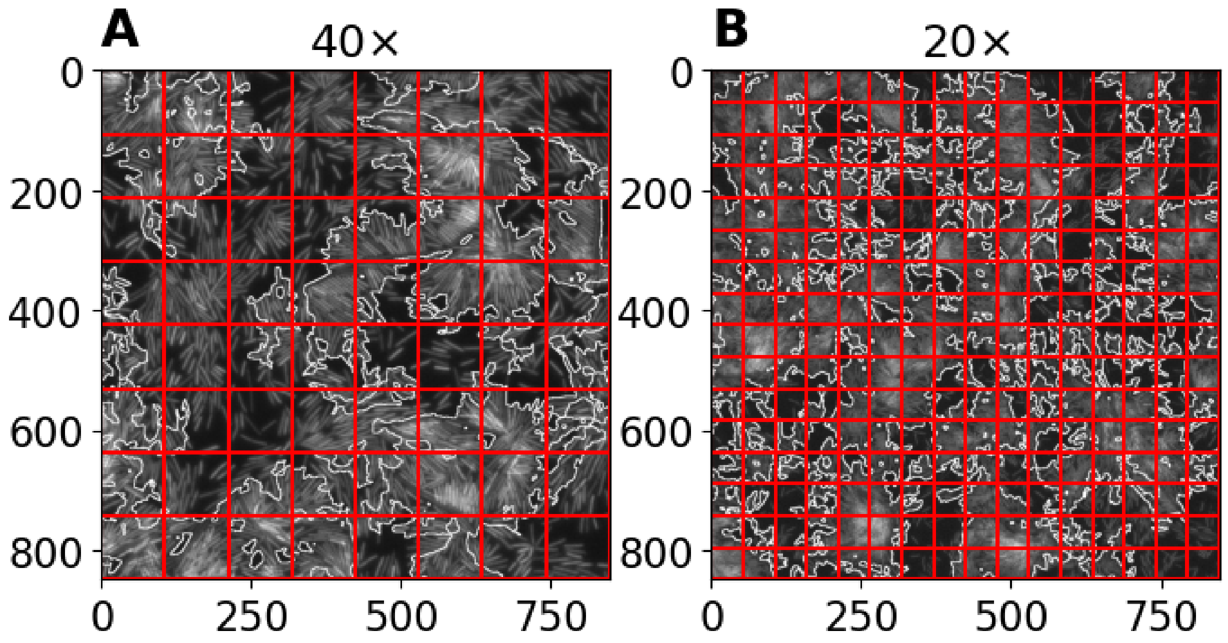

Figure S2: **Binning approach.** Bins superimposed on microscopic image with outlined stationary aggregates at **A** 40× magnification and **B** 20× magnification.

same time, where one image (left panel, see Fig. S3**A**) shows roughly 1% of the cells and the other image (right panel, see Fig. S3**B**) the remaining 99%. Hence, the position of the few cells in the left panel within the collective (right panel) can be obtained by merging the images. Trajectories of cells in the left panel were obtained by identifying cells via Hessian- and Frangi-filters (see Fig. S3**C**) and using the Crocker–Grier algorithm (*65*) implemented in trackpy (*66*) (see Fig. S3**D**).

We compute mean-square displacements (MSD) of individual trajectories $\langle r^2(\tau)\rangle = \langle |r(t) - r(t+\tau)|^2\rangle$, where $r(t)$ is the position of a cell at time t and the average is taken over all suitable times t. For large times one expects the relation $\langle r^2(\tau)\rangle \sim \tau^\beta$, where β indicates diffusive ($\beta \approx 1$), subdiffusive ($\beta \ll 1$) or superdiffusive ($\beta \gg 1$) behaviour. Based on a fit of the MSD to a power-law, we classify trajectories into the aforementioned categories. Furthermore, we introduce a transitive category to account for trajectories which fit poorly to a power-law, i.e. if the standard deviation of the fit of β is large. When fitting to an power-law, we log transform the τ and $\langle r^2(\tau)\rangle$ data and use linear regression to get the best linear approximation.

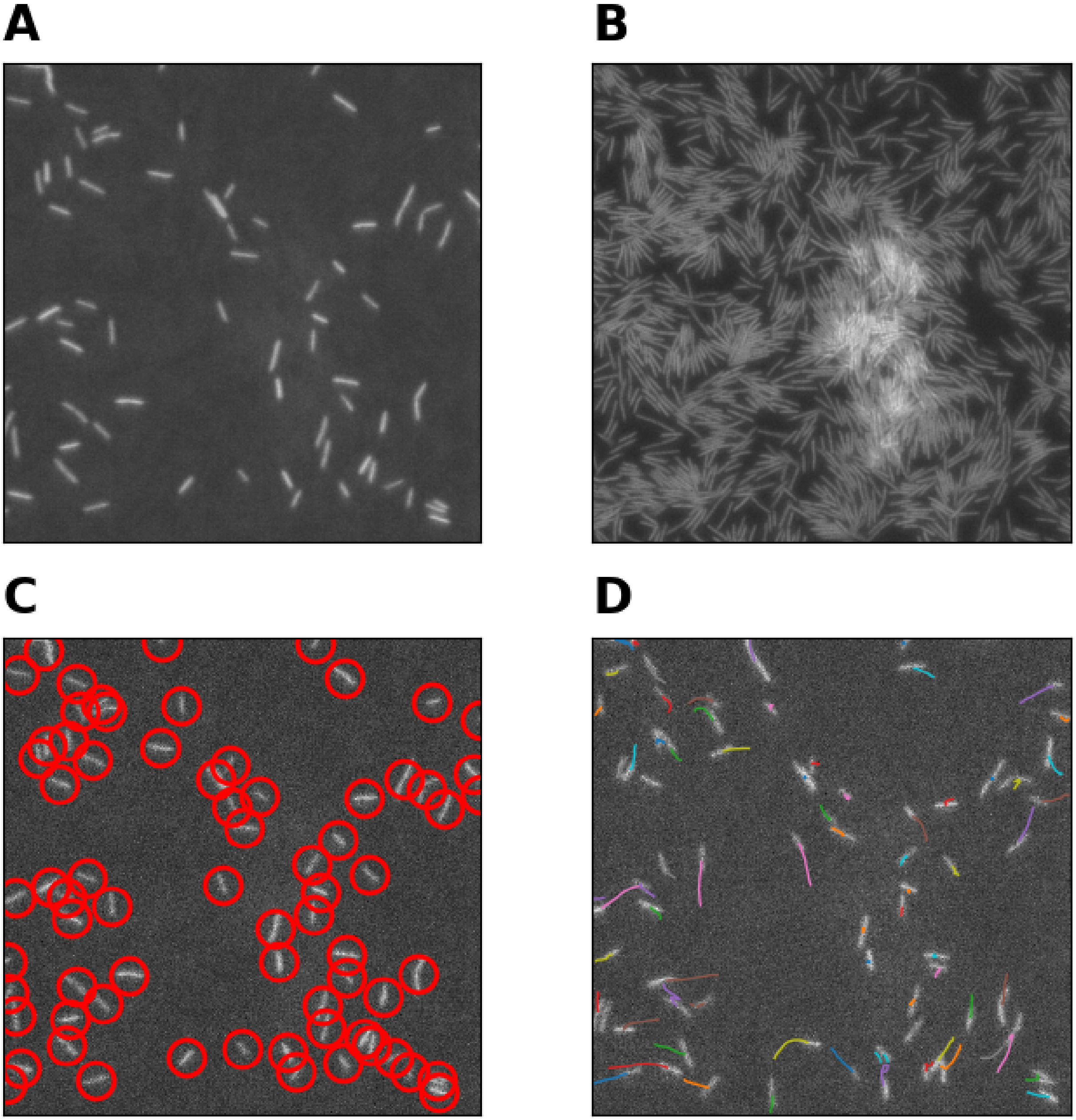

Figure S3: **Single-cell tracking.** **A** Left panel (see text), representing roughly 1% of all cells within the field of view. **B** Right panel (see text), representing the exact field of view at the same time and the remaining 99% of all cells within the field of view. **C** Identified cells from the left panel are encircled in red. **D** Trajectories of length 0.2 s obtained from the left panel superimposed on the microscopic image at $t = 0.2$ s. Each trajectory is represented by an individual color.

REFERENCES AND NOTES

1. R. Kolter, E. P. Greenberg, The superficial life of microbes. *Nature* **441**, 300–302 (2006).

2. D. B. Kearns, R. Losick, Cell population heterogeneity during growth of *Bacillus subtilis*. *Genes Dev.* **19**, 3083–3094 (2005).

3. D. López, R. Kolter, Extracellular signals that define distinct and coexisting cell fates in *Bacillus subtilis*. *FEMS Microbiol. Rev.* **34**, 134–149 (2010).

4. S. K. Desai, L. J. Kenney, Switching lifestyles is an in vivo adaptive strategy of bacterial pathogens. *Front. Cell. Infect. Microbiol.* **9**, 421 (2019).

5. S. Srinivasan, C. N. Kaplan, L. Mahadevan, A multiphase theory for spreading microbial swarms and films, *eLife* **8**, e42697 (2019).

6. I. Grobas, D. G. Bazzoli, M. Asally, Biofilm and swarming emergent behaviours controlled through the aid of biophysical understanding and tools. *Biochem. Soc. Trans.* **48**, 2903–2913 (2020).

7. L. Hall-Stoodley, J. W. Costerton, P. Stoodley, Bacterial biofilms: From the natural environment to infectious diseases. *Nat. Rev. Microbiol.* **2**, 95–108 (2004).

8. H. Vlamakis, Y. Chai, P. Beauregard, R. Losick, R. Kolter, Sticking together: Building a biofilm the *Bacillus subtilis* way. *Nat. Rev. Microbiol.* **11**, 157–168 (2013).

9. D. B. Kearns, R. LosickSwarming motility in undomesticated *Bacillus subtilis*. *Mol. Microbiol.* **49**, 581–590 (2003).

10. D. B. Kearns, A field guide to bacterial swarming motility. *Nat. Rev. Microbiol.* **8**, 634–644 (2010).

11. A. Be’er, G. Ariel, A statistical physics view of swarming bacteria. *Mov. Ecol.* **7**, 9 (2019).

12. S. B. Guttenplan, D. B. Kearns, Regulation of flagellar motility during biofilm formation. *FEMS Microbiol. Rev.* **37**, 849–871 (2013).

13. H. Jeckel, E. Jelli, R. Hartmann, P. K. Singh, R. Mok, J. F. Totz, L. Vidakovic, B. Eckhardt, J. Dunkel, K. Drescher,, Learning the space-time phase diagram of bacterial swarm expansion. *Proc. Natl. Acad. Sci. U.S.A.* **116**, 1489–1494 (2019).

14. A. Be'er, B. Ilkanaiv, R. Gross, D. B. Kearns, S. Heidenreich, M. Bär, G. Ariel, A phase diagram for bacterial swarming. *Commun. Phys.* **3**, 66 (2020).

15. D. Romero, C. Aguilar, R. Losick, R. Kolter, Amyloid fibers provide structural integrity to *Bacillus subtilis* biofilms. *Proc. Natl. Acad. Sci.* **107**, 2230–2234 (2010).

16. I. Grobas, M. Polin, M. Asally, Swarming bacteria undergo localized dynamic phase transition to form stress-induced biofilms, *eLife* **10**, e62632 (2021).

17. Y. Fily, M. C. Marchetti, Athermal phase separation of self-propelled particles with no alignment. *Phys. Rev. Lett.* **108**, 235702 (2012).

18. J. Bialké, H. Löwen, T. Speck, Microscopic theory for the phase separation of self-propelled repulsive disks. *Europhys. Lett.* **103**, 30008 (2013).

19. M. E. Cates, J. Tailleur, Motility-induced phase separation. *Annu. Rev. Condens. Matter Phys.* **6**, 219–244 (2015).

20. L. S. Cairns, V. L. Marlow, E. Bissett, A. Ostrowski, N. R. Stanley-Wall, A mechanical signal transmitted by the flagellum controls signalling in *Bacillus subtilis*. *Mol. Microbiol.* **90**, 6–21 (2013).

21. D. B. Kearns, F. Chu, R. Rudner, R. Losick, Genes governing swarming in *Bacillus subtilis* and evidence for a phase variation mechanism controlling surface motility. *Mol. Microbiol.* **52**, 357–369 (2004).

22. S. S. Branda, F. Chu, D. B. Kearns, R. Losick, R. Kolter, A major protein component of the *Bacillus subtilis* biofilm matrix. *Mol. Microbiol.* **59**, 1229–1238 (2006).

23. S. O. Bendori, S. Pollak, D. Hizi, A. Eldar, G. A. O'Toole, The RapP-PhrP quorum-sensing system of *Bacillus subtilis* strain NCIB3610 affects biofilm formation through multiple targets, due to an atypical signal-insensitive allele of RapP. *J. Bacteriol.* **197**, 592–602 (2015).

24. E. J. Murray, T. B. Kiley, N. R. Stanley-Wall, A pivotal role for the response regulator DegU in controlling multicellular behaviour. *Microbiology* **155**, 1–8 (2009).

25. Y. Chai, F. Chu, R. Kolter, R. Losick, Bistability and biofilm formation in *Bacillus subtilis*. *Mol. Microbiol.* **67**, 254–263 (2008).

26. Y. Chai, T. Norman, R. Kolter, R. Losick, An epigenetic switch governing daughter cell separation in *Bacillus subtilis*. *Genes Dev.* **24**, 754–765 (2010).

27. Y. Chai, R. Kolter, R. Losick, Paralogous antirepressors acting on the master regulator for biofilm formation in *Bacillus subtilis*. *Mol. Microbiol.* **74**, 876–887 (2009).

28. N. D. Lord, T. M. Norman, R. Yuan, S. Bakshi, R. Losick, J. Paulsson, Stochastic antagonism between two proteins governs a bacterial cell fate switch. *Science* **366**, 116–120 (2019).

29. K. M. Blair, L. Turner, J. T. Winkelman, H. C. Berg, D. B. Kearns, A molecular clutch disables flagella in the *Bacillus subtilis* biofilm. *Science* **320**, 1636–1638 (2008).

30. I. Buttinoni, J. Bialké, F. Kümmel, H. Löwen, C. Bechinger, T. Speck, Dynamical clustering and phase separation in suspensions of self-propelled colloidal particles. *Phys. Rev. Lett.* **110**, 238301 (2013).

31. D. Geyer, D. Martin, J. Tailleur, D. Bartolo, Freezing a flock: Motility-induced phase separation in polar active liquids. *Phys. Rev. X* **9**, 031043 (2019).

32. A. Sokolov, I. S. Aranson, J. O. Kessler, R. E. Goldstein, Concentration dependence of the collective dynamics of swimming bacteria. *Phys. Rev. Lett.* **98**, 158102 (2007).

33. G. Ariel, M. Sidortsov, S. D. Ryan, S. Heidenreich, M. Bär, A. Be'er, Collective dynamics of two-dimensional swimming bacteria: Experiments and models. *Phys. Rev. E* **98**, 032415 (2018).

34. G. Liu, A. Patch, F. Bahar, D. Yllanes, R. D. Welch, M. C. Marchetti, S. Thutupalli, J. W. Shaevitz, Self-driven phase transitions drive *Myxococcus xanthus* fruiting body formation. *Phys. Rev. Lett.* **122**, 248102 (2019).

35. X.-q. Shi, H. Chaté, Self-propelled rods: Linking alignment-dominated and repulsion-dominated active matter. arXiv:1807.00294 [cond-mat.soft] (1 July 2018).

36. R. van Damme, J. Rodenburg, R. van Roij, M. Dijkstra, Interparticle torques suppress motility-induced phase separation for rodlike particles. *J. Chem. Phys.* **150**, 164501 (2019).

37. R. Großmann, I. S. Aranson, F. Peruani, A particle-field approach bridges phase separation and collective motion in active matter. *Nat. Commun.* **11**, 5365 (2020).

38. V. M. Worlitzer, G. Ariel, A. Be’er, H. Stark, M. Bär, S. Heidenreich, Motility-induced clustering and meso-scale turbulence in active polar fluids. *New J. Phys.* **23**, 033012 (2021).

39. V. M. Worlitzer, G. Ariel, A. Be'er, H. Stark, M. Bär, S. Heidenreich, Turbulence-induced clustering in compressible active fluids. *Soft Matter* **17**, 10447–10457 (2021).

40. M. E. Cates, D. Marenduzzo, I. Pagonabarraga, J. Tailleur, Arrested phase separation in reproducing bacteria creates a generic route to pattern formation. *Proc. Natl. Acad. Sci. U.S.A.* **107**, 11715–11720 (2010).

41. T. Grafke, M. E. Cates, E. Vanden-Eijnden, Spatiotemporal self-organization of fluctuating bacterial colonies. *Phys. Rev. Lett.* **119**, 188003 (2017).

42. G. Farnebäck, Two-frame motion estimation based on polynomial expansion. *Lect. Notes Comput. Sci.* **2749**, 363 (2003).

43. R. McElreath, *Statistical Rethinking: A Bayesian Course with Examples in R and Stan* (Chapman and Hall/CRC, 2020).

44. H. P. Zhang, A. Be’er, E.-L. Florin, H. L. Swinney, Collective motion and density fluctuations in bacterial colonies. *Proc. Natl. Acad. Sci. U.S.A.* **107**, 13626–13630 (2010).

45. F. Peruani, J. Starruß, V. Jakovljevic, L. Søgaard-Andersen, A. Deutsch, M. Bär, Collective motion and nonequilibrium cluster formation in colonies of gliding bacteria. *Phys. Rev. Lett.* **108**, 098102 (2012).

46. J. Starruß, F. Peruani, V. Jakovljevic, L. Søgaard-Andersen, A. Deutsch, M. Bär, Pattern-formation mechanisms in motility mutants of *Myxococcus xanthus*. *Interface Focus* **2**, 774–785 (2012).

47. F. Peruani, M. Bär, A kinetic model and scaling properties of non-equilibrium clustering of self-propelled particles. *New J. Phys.* **15**, 065009 (2013).

48. G. Ariel, A. Rabani, S. Benisty, J. D. Partridge, R. M. Harshey, A. Be'er, Swarming bacteria migrate by Lévy Walk. *Nat. Commun.* **6**, 8396 (2015).

49. G. Ariel, A. Be'er, A. Reynolds, Chaotic model for Lévy walks in swarming bacteria.. *Phys. Rev. Lett.* **118**, 228102 (2017)

50. A. Kaiser, H. H. Wensink, H. Löwen, How to capture active particles. *Phys. Rev. Lett.* **108**, 268307 (2012).

51. P. Pietzonka, E. Fodor, C. Lohrmann, M. E. Cates, U. Seifert, Autonomous engines driven by active matter: Energetics and design principles. *Phys. Rev. X* **9**, 041032 (2019).

52. H. Vlamakis, C. Aguilar, R. Losick, R. Kolter, Control of cell fate by the formation of an architecturally complex bacterial community. *Genes Dev.* **22**, 945–953 (2008).

53. J. van Gestel, H. Vlamakis, R. Kolter, From cell differentiation to cell collectives: *Bacillus subtilis* uses division of labor to migrate. *PLoS Biol.* **13**, e1002141 (2015).

54. J. van Gestel, H. Vlamakis, R. Kolter, Division of labor in biofilms: The ecology of cell differentiation. *Microbiol. Spectr.* **3**, MB-0002-2014 (2015).

55. N. Verstraeten, K. Braeken, B. Debkumari, M. Fauvart, J. Fransaer, J. Vermant, J. Michiels, Living on a surface: Swarming and biofilm formation. *Trends Microbiol.* **16**, 496–506 (2008).

56. G. C. L. Wong, J. D. Antani, P. P. Lele, J. Chen, B. Nan, M. J. Kühn, A. Persat, J. L. Bru, N. M. Høyland-Kroghsbo, A. Siryaporn, J. C. Conrad, F. Carrara, Y. Yawata, R. Stocker, Y. V Brun, G. B. Whitfield, C. K. Lee, J. de Anda, W. C. Schmidt, R. Golestanian, G. A. O'Toole, K. A. Floyd, F. H. Yildiz, S. Yang, F. Jin, M. Toyofuku, L. Eberl, N. Nomura, L. A. Zacharoff, M. Y. el-Naggar, S. E. Yalcin, N. S. Malvankar, M. D. Rojas-Andrade, A. I. Hochbaum, J. Yan, H. A. Stone, N. S. Wingreen, B. L. Bassler, Y. Wu, H. Xu, K. Drescher, J. Dunkel, Roadmap on emerging concepts in the physical biology of bacterial biofilms: From surface sensing to community formation. *Phys. Biol.* **18**, 051501 (2021).

57. Y. Fu, H. Yu, X. Zhang, P. Malgaretti, V. Kishore, W. Wang, Microscopic swarms: From active matter physics to biomedical and environmental applications. *Micromachines* **13**, 295 (2022).

58. J. D. Partridge, R. M. Harshey, Swarming: Flexible roaming plans. *J. Bacteriol.* **195**, 909–918 (2013).

59. S. Rütschlin, T. Böttcher, Inhibitors of bacterial swarming behavior. *Chem. A Eur. J.* **26**, 964–979 (2020).

60. S. C. Takatori, K. K. Mandadapu, Motility-induced buckling and glassy dynamics regulate three-dimensional transitions of bacterial monolayers. arXiv:2003.05618 [cond-mat.soft] (12 March 2020).

61. S. Guiziou, V. Sauveplane, H.-J. Chang, C. Clerté, N. Declerck, M. Jules, J. Bonnet, A part toolbox to tune genetic expression in *Bacillus subtilis*. *Nucleic Acids Res.* **44**, 7495–7508 (2016).

62. S. Peled, S. D. Ryan, S. Heidenreich, M. Bär, G. Ariel, A. Be'er, Heterogeneous bacterial swarms with mixed lengths. *Phys. Rev. E* **103**, 032413 (2021).

63. B.-M. Koo, G. Kritikos, J. D. Farelli, H. Todor, K. Tong, H. Kimsey, I. Wapinski, M. Galardini, A. Cabal, J. M. Peters, A. B. Hachmann, D. Z. Rudner, K. N. Allen, A. Typas, C. A. Gross, Construction and analysis of two genome-scale deletion libraries for *Bacillus subtilis*. *Cell Syst.* **4**, 291–305.e7 (2017).

64. G. Bradski, The openCV library, *Dr. Dobb's Journal of Software Tools for the Professional Programmer* **25**, 120–125 (2000).

65. J. C. Crocker, D. G. Grier, Methods of digital video microscopy for colloidal studies. *J. Colloid Interface Sci.* **179**, 298–310 (1996).

66. D. B. Allan, T. Caswell, N. C. Keim, C. M. van der Wel, R. W. Verweij (2021); https://zenodo.org/record/4682814#.YoKx1lTP1D8.
